# Cap-Assisted Endoscopic Mucosal Resection of an Incidental Rectal Traumatic Neuroma

**DOI:** 10.1155/2019/8328456

**Published:** 2019-10-27

**Authors:** Elias Estifan, Varun Patel, Matthew Grossman

**Affiliations:** ^1^Department of Internal Medicine, St. Joseph's University Medical Center-New York Medical College, Paterson, NJ 07503, USA; ^2^Division of Gastroenterology, St. Joseph's University Medical Center-New York Medical College, Paterson, NJ 07503, USA

## Abstract

A traumatic neuroma is a well-known complication after acute trauma to a peripheral nerve; the nerve tries to re-establish continuity by an orderly growth of axons from the peripheral to distal stump through the proliferation of Schwann cells. However, this process is not always perfect, and aberrant repair gives rise to a neuroma. We present a 50-year-old female who underwent an initial colonoscopy for change in bowel habits and was found to have a 7 mm submucosal lesion in the proximal rectum. Endoscopic ultrasound was done which showed a hypoechoic lesion in the submucosal plane without muscularis propria invasion. The patient underwent successful cap-assisted endoscopic mucosal resection of the lesion without complication. Pathology of the specimen revealed a traumatic rectal neuroma with immunostaining positive for S100. However, this patient did not have any known risk factors such as previous surgery including polypectomy or hemorrhoidectomy or any previous rectal manipulation. Interestingly, this is the second case of traumatic rectal neuroma reported in the English-language literature.

## 1. Introduction

A traumatic neuroma develops from a non-neoplastic proliferation of the proximal end of a severed, partially transected, or injured nerve as a result of trauma or surgery. Most commonly, the lesion demonstrates pain whenever palpated or manipulated. The most common location for a traumatic neuroma is the lower extremity after an amputation, followed by the head and the neck area, especially the oral cavity after a tooth extraction [1]. We are presenting a rare case of a traumatic rectal neuroma, which was excised by cap-assisted endoscopic mucosal resection. Only one other case has been reported at this anatomic site in our English literature review [2].

## 2. Case Presentation

**Figure 1:**
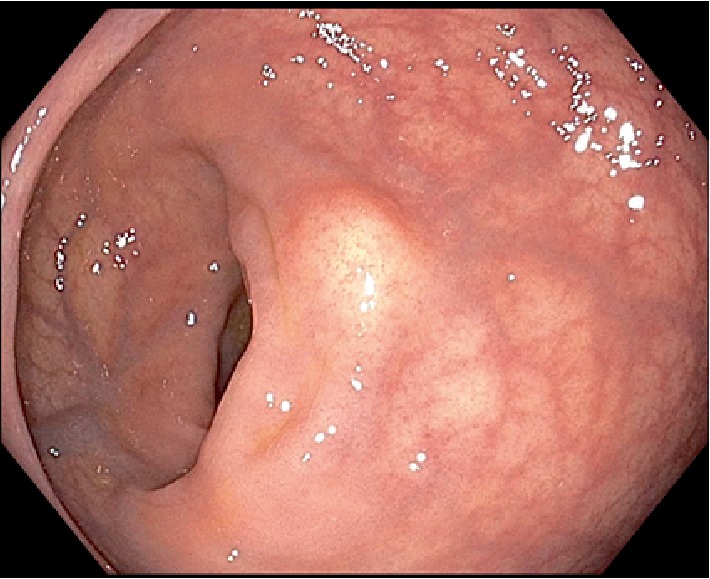
Endoscopic image showing a submucosal nodule in the rectum at the 3 o'clock position located 12 cm from the anal verge.

**Figure 2:**
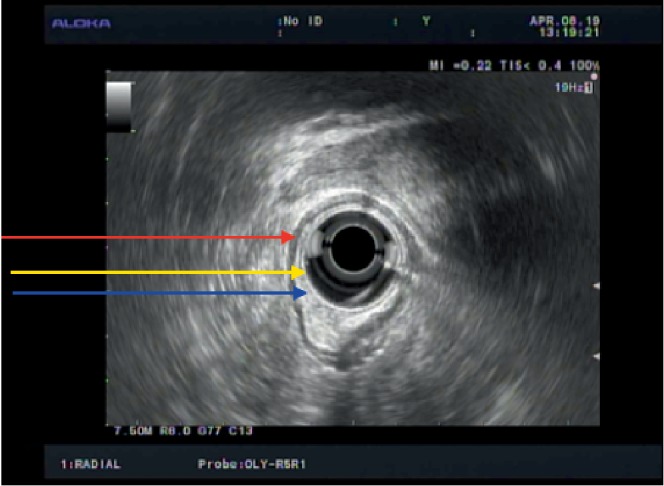
Rectal endoscopic ultrasound (EUS) showing a localized hypoechoic submucosal lesion with tiny hyperechoic bands without muscularis propria invasion. Muscularis mucosae (yellow arrow), submucosa (blue arrow), and muscularis propria (red arrow).

**Figure 3:**
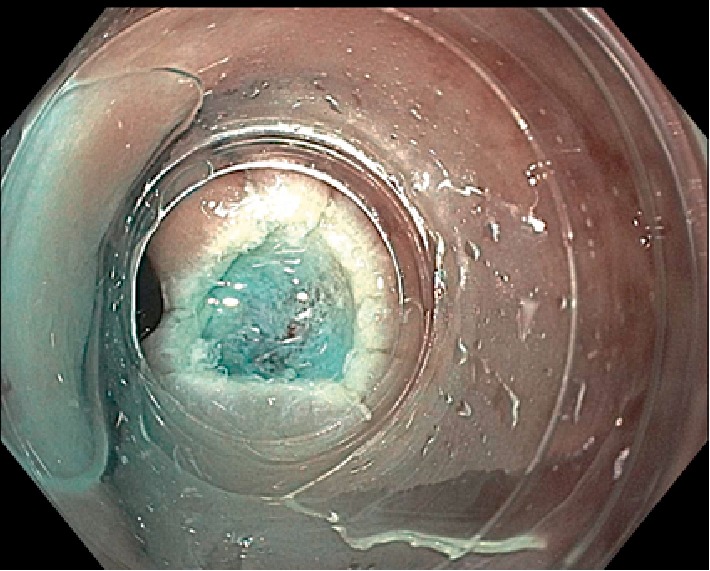
Endoscopic image showing *en-bloc* cap-assisted endoscopic mucosal resection of the rectal lesion.

**Figure 4:**
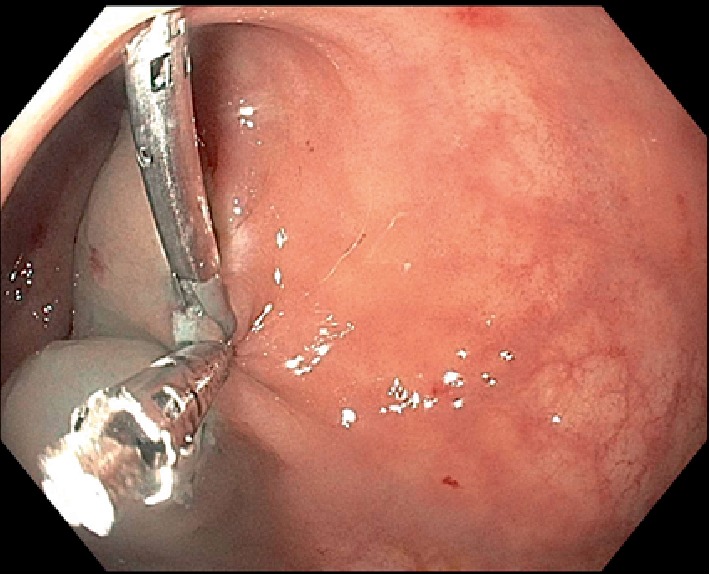
Endoscopic image showing successful closure of the rectal mucosal resection with two hemostatic clips.

**Figure 5:**
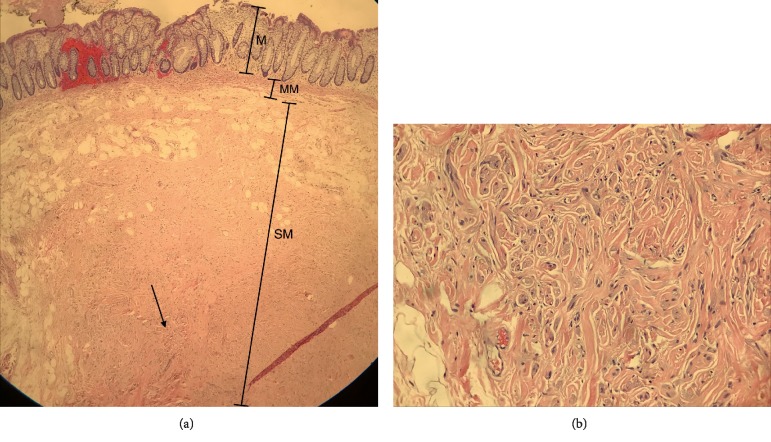
Pathology images of the traumatic rectal neuroma. (a) Low-power view. Note the mucosa on the top (M), the muscularis mucosae (MM), and the submucosa (SM). The lesion contained in the submucosal (arrow). (b) High-power view of the neural cells in the submucosa comprised of haphazardly proliferating spindle cells.

**Figure 6:**
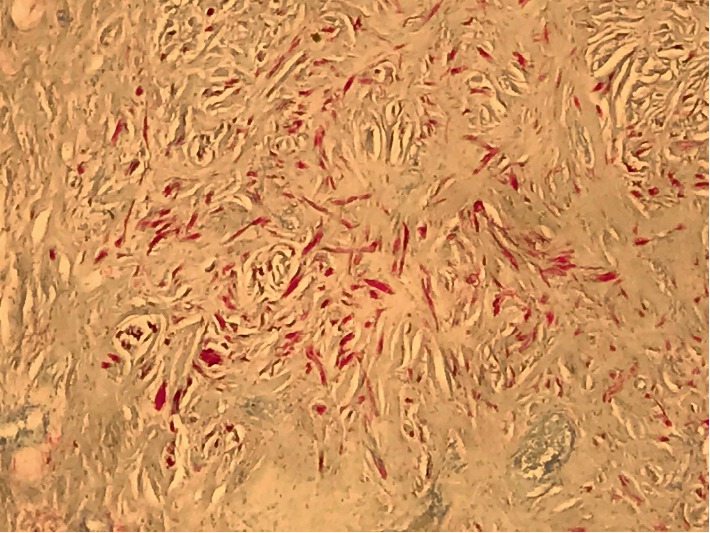
Pathology images of Immunostaining for S100, which including spindle and epithelioid neuro cells.

A 50-year-old female with a past medical history of asthmatic bronchitis underwent an initial colonoscopy procedure after complaining of 3 weeks of loose nonbloody bowel movements. The patient denied any rectal pain or prior rectal manipulation. On colonoscopy, an incidental 7 mm submucosal nodule was seen in the proximal rectum at 12 cm from the anal verge (Figure 1). The initial endoscopic biopsies taken from this area, however showed unremarkable rectal mucosa on histology. She then underwent an endoscopic ultrasound (EUS) of the localized submucosal lesion located 12 cm from the anal verge, at the 3 o'clock position (Figure 2). The lesion was homogenously hypoechoic, measuring up to 13 mm in thickness. The lesion was contained within the submucosal plane without muscularis propria invasion. The decision was made to pursue a cap-assisted endoscopic mucosal resection with an adult endoscope. A Carr-locke needle was used to inject approximately 5 ml of a mixture of saline and methylene blue, which provided an adequate lift of the lesion from the muscularis propria layer. An *en bloc* endoscopic mucosal resection with a cap and snare was achieved (Figure 3). Two hemostatic clips were placed to close the mucosal defect (Figure 4). The resected specimen was examined, showing no muscular fibers. Pathology of the specimen revealed a well-circumscribed 4 mm submucosal traumatic neuroma comprised of haphazardly proliferating spindle cells consistent with Schwann cells embedded in a fibrous stroma (Figure 5). Immunostaining was positive for S100 (Figure 6). The patient tolerated the procedure well and had no complications on one-month follow-up.

## 3. Discussion

Neuromas are benign neural proliferations that occur after nerve injury. A neuroma can occur after a laceration which results in micro-trauma of peripheral nerves from stretching or compression of local tissues and can arise 1–12 months after transection or injury [1, 3, 4]. Transection of the peripheral nerve will cause loss of essential macromolecules and stimulate the distal portion of the nerve to undergo Wallerian degeneration followed by regeneration of the nerve by expression of growth factors at the transection site of Schwann cell. This process, however, is not always perfect, as it can grow aberrantly, forming dense nerve tangles called neuromas. Extending the distance between two injured nerve segments or an absence of a distal segment could result in a haphazard growth [5–8].

Traumatic neuromas are divided into spindle neuroma and terminal neuroma. A spindle neuroma is characterized by an internal, focal, fusiform swelling secondary to constant friction or irritation due to a nondisrupted, injured, but intact nerve trunk [1]. A terminal neuroma is the result of severe trauma with partial avulsion, disruption, or total transection of a nerve [1]. Histologically, it corresponds as a non-neoplastic, nonencapsulated tangled masses of axons, Schwann cells, endoneurial cells, and perineurial cells in a dense collagenous matrix with surrounding fibroblasts [1]. On high-resolution endoscopic ultrasound (EUS), a neuroma appears homogenously hypoechoic, sometimes with small hyperechoic internal bands. Our patient's EUS demonstrated that the lesion was contained to the submucosal layer, giving rise to a differential diagnosis of lipoma, carcinoid, or ectopic pancreas. However, the stains were negative for these pathologies.

In rat models, it has been demonstrated that inhibiting nerve growth factor (NGF) following nerve injury could reduce neuroma formation and neuropathic pain without damaging the cell bodies [5]. Therefore, management of traumatic neuroma in extremities consists of steroid injections, nerve stimulation and/or physical therapy, however half of the patients will ultimately require surgical resection for definitive management [1, 2, 6]. There are no clear established recommendations for treating a traumatic rectal neuroma; however, as shown in our case, using cap-assisted endoscopic mucosal resection (EMR) technique was safe and successful, achieving *en bloc* resection. Nevertheless, the risk of recurrence has been reported to be high, and therefore, surveillance colonoscopy should be recommended.

The usual presentation of a traumatic neuroma is of pain; however, our patient presented asymptomatically. This can possibly be attributed to the lack of somatic innervation within the rectal submucosa as opposed to neuromas located in an extremity. In the only other case report of a traumatic rectal neuroma, the authors hypothesized the lesion likely arose as a result of trauma to the submucosal Meissner's plexus fibers or Auerbach's plexus fibers after a previous polypectomy [2]. Interestingly, our patient never had a previous colonoscopy, rectal trauma/manipulation or anal receptive intercourse, which could have precipitated a neuroma. To our knowledge, this is the second case of a traumatic rectal neuroma, however the first case of incidental traumatic rectal neuroma was reported in the English-language literature.
